# Quantification of coral sperm collected during a synchronous spawning event

**DOI:** 10.7717/peerj.2180

**Published:** 2016-07-14

**Authors:** Aaron Teo, James R. Guest, Mei Lin Neo, Kareen Vicentuan, Peter A. Todd

**Affiliations:** 1 Department of Biological Sciences, National University of Singapore, Singapore; 2 SECORE International, Johor, Malaysia; 3 Tropical Marine Science Institute, National University of Singapore, Singapore; 4 St John’s Island Marine Laboratory, National University of Singapore, Singapore

**Keywords:** Eggs, Coral, Gametes, Model, Sperm, Reproduction

## Abstract

Most studies of coral reproductive biology to date have focused on oocyte numbers and sizes. Only one (*ex situ*) study has enumerated sperm numbers, even though these data have multiple potential applications. We quantified total coral sperm and eggs per gamete bundle collected from six species *in situ* during a synchronous spawning event in Singapore. Egg-sperm bundles were captured midwater as they floated towards the surface after being released by the colony. For each sample, a semi-transparent soft plastic bottle was squeezed and released to create a small suction force that was used to ‘catch’ the bundles. This technique provided several advantages over traditional methods, including low cost, ease of use, no diving prior to the night of collection needed, and the ability to target specific areas of the colony. The six species sampled were *Echinophyllia aspera*, *Favites abdita*, *F. chinensis*, *Merulina ampliata, M. scabricula* and *Platygyra pini*. The mean number of sperm packaged within one egg-sperm bundle ranged from 2.04 × 10^6^ to 1.93 × 10^7^. The mean number of eggs per egg-sperm bundle ranged from 26.67 (SE ± 3.27) to 85.33 (SE ± 17.79). These data are critical for fertilisation success models, but the collection technique described could also be applied to studies requiring *in situ* spawning data at the polyp level.

## Introduction

Approximately 86% of scleractinian coral species with documented reproductive traits have been identified as broadcast spawners (Baird, Guest & Willis, 2009). This mode of reproduction is prevalent in many aquatic taxa across multiple phyla (Levitan, 1998) and involves the shedding of gametes into the water column where external fertilisation and embryogenesis can occur (Szmant, 1986; Giese & Kanatani, 1987; Wray, 1995). The majority of broadcast spawning corals release simultaneously both eggs and sperm packaged together in buoyant egg-sperm bundles during a spawning event (Babcock & Heyward, 1986). These egg-sperm bundles aggregate on the surface, concentrating the gametes to ensure that fertilisation can occur in a small space relative to the entire three-dimensional seascape (Moláček, Denny & Bush, 2012).

Due to the multi-specific synchronous nature of many spawning events it is practically impossible to collect gametes from a particular species or colony from within spawn slicks. Hence, studies that involve coral gamete collection have traditionally obtained spawned samples by trapping them before they reach the surface using nets placed directly over colonies (Guest et al., 2010) or by collecting them *ex situ* from colonies that have been moved to aquaria prior to spawning (Negri & Heyward, 2001). An alternative technique of collecting gamete data is via the histological analysis of polyp sections collected from excised coral fragments (Nozawa & Lin, 2014). As the development of eggs can be monitored within the ovaries, giving estimates of a polyp’s fecundity, this approach has revealed abundant information regarding coral gametogenesis, spawning periods and synchronicity cues (Manfred & Veghel, 1994; Vermeij et al., 2004), as well as mature oocyte sizes (Nozawa & Lin, 2014).

There is, however, a dearth of information on coral sperm compared to oocytes. Most studies focus on spermatogenesis using histological analyses (Goffredo, Telò & Scanabissi, 2000) to elucidate the impacts of anthropogenic disturbances such as sedimentation (Gilmour, 1999; Humphrey et al., 2008; Jones, Ricardo & Negri, 2015), heavy metal pollution (Reichelt-Brushett & Harrison, 1999; Negri & Heyward, 2001; Reichelt-Brushett & Harrison, 2005; Victor & Richmond, 2005) and ocean acidification (Morita et al., 2010). Others have examined factors that influence fertilisation success (Oliver & Babcock, 1992), for example, changes in sperm motility due to increased acidity (Morita et al., 2010) as well as damage and clumping due to suspended sediment (Ricardo et al., 2015). Sperm data, such as the amount released during a spawning, are mandatory in simulation models that attempt to make realistic predictions of sperm dilution and fertilisation rates (Babcock, Mundy & Whitehead, 1994; Metaxas, Scheibling & Young, 2002). Such models allow for general or site-specific estimates of a population’s reproductive output—complementing other forms of data used in reef health monitoring and assessment, e.g. life tables and recruitment rates, thereby contributing to conservation management plans.

Most notably, other than for *Acropora digitifera* (Paxton et al., 2015), there is a conspicuous absence of data regarding the number of sperm released by scleractinian corals during spawning. Hence, the main aim of the present study was to quantify the amount of sperm within a coral gamete bundle through *in situ* sampling of various coral species during a synchronous spawning event using a novel yet simple technique of collecting small samples of intact coral gamete bundles in midwater.

## Materials and Methods

### Study site

Pulau Satumu (Raffles Lighthouse) is a small rocky island measuring only 1.3 ha. Due to the relatively strong currents and distance (∼14 km) from Singapore’s main island, the reefs around Pulau Satumu are less affected by chronic sedimentation compared to other local reefs. Multi-species coral spawning events occur during the week following the March or April full moon (Guest et al., 2005).

### Gamete collection

Coral gamete bundles were collected during the spawning events that occurred on the nights of 19^th^ and 20^th^ April 2014 between 20.00–22.00 h from the fringing reefs to the west of Pulau Satumu. Opportunistic sampling within a 50 × 5 m^2^ belt transect was conducted by identifying colonies that were ready to spawn (by observing gamete bundles “setting” just below the polyp mouth) and waiting for the release of the gametes.

Using an underwater digital camera, each selected colony was photographed with a uniquely labeled transparent ziplock bag placed alongside it so that the bags could later be matched to the coral species. Semi-transparent 30 ml soft plastic bottles (outer dimensions: 59 × 27 mm, length × diameter; 10 mm diameter nozzle opening) were used to collect gamete bundles. The bottle was squeezed and released to create a small suction force that was used to ‘catch’ bundles as they floated towards the surface after being released by the colony. This technique enabled the capture of intact gamete bundles from specific colonies. The bottles were handled carefully to avoid breaking the bundles prematurely. On the first day three bundles were captured within each bottle from five species whereas on the second day six bundles were captured within one bottle from one species.

Three bottles of samples were collected per colony and placed in their colony-specific ziplock bags for transportation back to the boat. The intact gamete bundles and seawater contained within each bottle were transferred into labeled falcon tubes prefilled with sufficient formaldehyde to produce 50 ml of solution at 3.7% concentration. The falcon tubes were then agitated by hand to break apart the gamete bundles and fix the eggs and sperm released.

### Gamete quantification

After the eggs were filtered out using an 80 μm sieve, three 10 μl aliquots were extracted per (agitated) falcon tube with a micropipette. Each aliquot was stained with 10 μl of trypan blue and left to stand for 15 min before the contents of the aliquot was injected into a Neubauer haemocytometer for counting. Sperm were visually distinguished from other matter in the solution by the distinctive shape of the sperm heads. All sperm within the 25 large squares (0.1 mm^3^ total volume) in the center grid were counted. The filtered eggs for each falcon tube were photographed and then counted using ImageJ (ImageJ 1.48 v; National Institutes of Health, Bethesda, MD, USA).

## Results and Discussion

**Figure 1:**
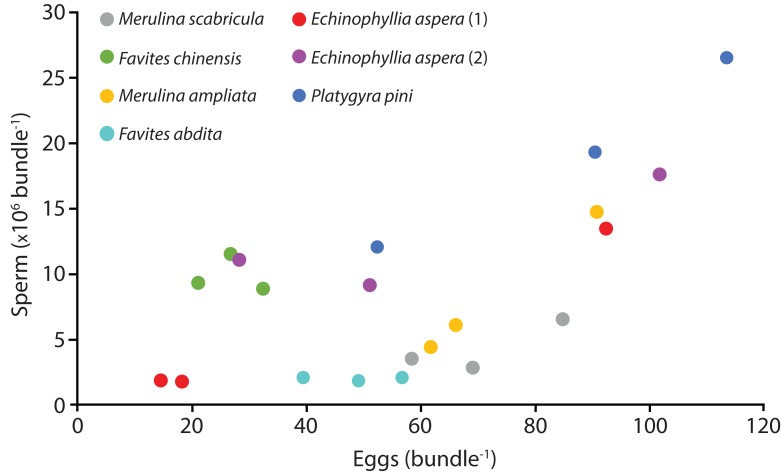
Number of sperm per bundle for the seven colonies sampled in April 2014. Each point represents the mean values for three egg-sperm bundles collected in one bottle (six egg-sperm bundles per bottle for *Echinophyllia aspera*). Three bottles were collected per colony. Two colonies were sampled for *E. aspera* (labelled 1 and 2); for all other species only one colony was sampled.

Six species (*Echinophyllia aspera*, *Favites abdita*, *F. chinensis*, *Merulina ampliata*, *M. scabricula*, and *Platygyra pini*) were sampled successfully. The mean number of sperm packaged within one egg-sperm bundle ranged from 2.04 × 10^6^ to 1.93 × 10^7^. The greatest number of sperm was released by *P. pini*, whereas the least was from *F. abdita* (Table 1). The mean (± SE) number of eggs per egg-sperm bundle varied from 26.67 (SE ± 3.27) to 85.33 (SE ± 17.79). *F. chinensis* had the highest sperm: egg ratio whereas *F. abdita* had the lowest (Table 1). There was generally a positive relationship between the number of eggs and number of sperm per bundle (Fig. 1).

**Table 1:** Mean (± SE) number of eggs and sperm per bundle for the seven colonies sampled in April 2014. Three samples (bottles) of egg-sperm bundles were collected per colony. Two colonies were sampled for *Echinophyllia aspera* (labelled 1 and 2); for all other species only one colony was sampled.

Species	Date	No of bundles in sample	Mean eggs (bundle^−1^)	Mean sperm (× 10^6^ bundle^−1^)	Sperm-egg ratio
*Favites abdita*	19/04	3	48.33 ± 5.01	2.04 ± 0.30	4.2 (× 10^4^):1
*F. chinensis*	19/04	3	26.67 ± 3.27	9.33 ± 1.62	35.0 (× 10^4^):1
*Merulina ampliata*	19/04	3	72.78 ± 9.03	8.44 ± 2.03	11.6 (× 10^4^):1
*M. scabricula*	19/04	3	70.67 ± 7.65	4.33 ± 0.72	6.1 (× 10^4^):1
*Platygyra pini*	19/04	3	85.33 ± 17.79	19.33 ± 2.85	22.7 (× 10^4^):1
*Echinophyllia aspera* (colony 1)	20/04	6	41.67 ± 25.36	5.76 ± 2.37	13.8 (× 10^4^):1
*E. aspera* (colony 2)	20/04	6	60.27 ± 21.72	12.63 ± 1.31	21.0 (× 10^4^):1

The technique of using small transparent soft plastic bottles as collection devices was effective at capturing individual gamete bundles and was simple enough to be used one-handed (freeing up the other hand to hold a torch, for example). Unlike the traditional approaches of using traps positioned above selected specimens or collecting entire colonies and moving them to tanks on land or on a boat, our technique required little preparation and no prior diving to set up traps or collect colonies. No aquarium facilities were required and no damage was done to donor colonies. Critically, the technique enables the direct quantification of sperm released during actual spawning. While this information may be extracted from egg-sperm bundles captured in traps positioned over colonies, or with nets swept by hand, there remains the possibility that these become broken up. Variation in polyp fecundity due to polyp position (Nozawa & Lin, 2014) is also not distinguishable when using trapped samples (as they are mixed during collection).

We required sperm quantity data for an ongoing modelling project to predict the fertilisation success of synchronous spawning corals and such data are likely to be important for other contemporary and future fertilisation models. However, additional information could be extracted from sperm collected using this technique. For example, sperm number and stage of development is usually described by the size of the spermaries present in tissue samples (Nozawa & Lin, 2014) without any quantification of the number of competent sperm present. The scarcity of sperm count data can hinder other aspects of coral reproduction research, including assessing the risks of sperm limitation and its antithesis, polyspermy, as well as the reproductive strategies that may have evolved to cope with these potential issues. Morphological data for sperm are also scarce, with few studies detailing their characteristics (Harrison, 1985; Steiner, 1991; Hagedorn et al. 2006) despite the possibility that these can yield information on sexual reproduction patterns (Harrison, 1985).

There was substantial variation in the average number of gametes per bundle sampled, with differences of up to ten times more sperm between samples recorded (after accounting for the number of bundles per sample). These differences were found across all levels, ranging from species to individual colonies and even individual samples of bundles from the same colony. The observed differences are likely to be due to a combination of factors such as species and genotypic differences in reproductive output, as well as the influence of age (Sakai, 1998), colony and polyp size (Hall & Hughes, 1996; Sakai, 1998; Nozawa & Lin, 2014) and polyp position within the colony (Sakai, 1998; Nozawa & Lin, 2014). Future studies could use the technique described here to target specific polyps in a colony to better understand intra-colonial variation in gamete output. Although this can be achieved via histology (and the breaking off of fragments from around the colony) not all gametes may be released during spawning as they can reabsorbed by the polyp (Harrison & Wallace, 1990), providing erroneous data. As the technique is non-destructive, repeatedly examining the same polyp also becomes a possibility.

As the threat of extinction looms over a third of all reef-building coral species (Carpenter et al., 2008; Plaisance et al., 2011), the need to improve the efficiency and effectiveness of management and restoration plans is increasingly urgent. Reproductive output data are critical to such conservation efforts; however, existing knowledge gaps hinder coral fertilisation models and the ability to predict the impact of disturbances. Our study introduces a simple, effective and non-destructive means of sampling intact egg-sperm bundles during coral spawning in the field. The technique helped elucidate the number of sperm packaged within an egg-sperm bundle and can potentially capture polyp-level variation in fecundity.

## Supplemental Information

10.7717/peerj.2180/supp-1 Supplemental Information 1 Raw data of sperm and egg counts for six coral species.

## Additional Information and Declarations

### Data Deposition

The following information was supplied regarding data availability: The raw data has been supplied as Supplemental Dataset Files.
