# Supplementary material for: Quantification of coral sperm collected during a synchronous spawning event
Source: PeerJ. 2016 Jul 14;4:e2180. doi: 10.7717/peerj.2180 (PMC4950549; doi:10.7717/peerj.2180)
Supplement: Supplemental Information 1 [file peerj-04-2180-s001.docx]

Table S1. Raw data of sperm counts for six coral species. Each 10 µl subsample was diluted with an equal volume of trypan blue before counting in a haemocytometer.

| Species | Bottle 1 subsamples | | | Bottle 2 subsamples | | | Bottle 3 subsamples | | |
| --- | --- | --- | --- | --- | --- | --- | --- | --- | --- |
|  | 1 | 2 | 3 | 1 | 2 | 3 | 1 | 2 | 3 |
| *Favites abdita* | 12 | 5 | 2 | 5 | 7 | 5 | 6 | 7 | 6 |
| *F. chinensis* | 36 | 25 | 19 | 35 | 34 | 35 | 4 | 12 | 52 |
| *Merulina ampliata* | 66 | 36 | 31 | 30 | 12 | 13 | 20 | 14 | 6 |
| *M. scabricula* | 6 | 3 | 17 | 21 | 22 | 16 | 11 | 9 | 12 |
| *Platygyra pini* | 64 | 40 | 70 | 31 | 45 | 33 | 104 | 46 | 89 |
| *Echinophyllia aspera* ( colony 1) | 9 | 12 | 13 | 10 | 9 | 14 | 56 | 137 | 51 |
| *E. aspera* (colony 2) | 51 | 53 | 62 | 105 | 114 | 98 | 64 | 73 | 62 |

Table S2. Raw data of egg counts for six coral species

| Species | Bottle 1 full sample | Bottle 2 full sample | Bottle 3 full sample |
| --- | --- | --- | --- |
| *Favites abdita* | 170 | 147 | 118 |
| *F. chinensis* | 97 | 80 | 63 |
| *Merulina ampliata* | 272 | 198 | 185 |
| *M. scabricula* | 207 | 254 | 175 |
| *Platygyra pini* | 271 | 157 | 340 |
| *Echinophyllia aspera* ( colony 1) | 87 | 109 | 554 |
| *E. aspera* (colony 2) | 306 | 610 | 169 |
